# Gene expression evaluation of antioxidant enzymes in patients with hepatocellular carcinoma: RT-qPCR and bioinformatic analyses

**DOI:** 10.1590/1678-4685-GMB-2019-0373

**Published:** 2021-04-02

**Authors:** Andressa de Freitas Alves, Ana Carolina de Moura, Huander Felipe Andreolla, Ana Beatriz Gorini da Veiga, Marilu Fiegenbaum, Márcia Giovenardi, Silvana Almeida

**Affiliations:** 1 Universidade Federal de Ciências da Saúde de Porto Alegre, Programa de Pós-graduação em Biociências, Porto Alegre, RS, Brazil.; 2 Universidade Franciscana, Santa Maria, RS, Brazil.

**Keywords:** Hepatocellular carcinoma, selenoproteins, antioxidant enzymes, oxidative stress, gene expression

## Abstract

Any condition leading to chronic liver disease is a potential oncogenic agent for hepatocellular carcinoma (HCC). Alterations in the expression of antioxidant enzymes could alter the redox balance. Our aim was to evaluate the expression of the genes GPX1, GPX4, SEP15, SELENOP, SOD1, SOD2, GSR, CAT, and NFE2L2 in patients with HCC. Differential gene expression analysis was performed using RNA-Seq data from the TCGA and GTEx databases, and RT-qPCR data from HCC patient samples. Bioinformatic analysis revealed significant differential expression in most genes. GPX4 expression was significantly increased (p=0.02), while SOD2 expression was significantly decreased (p=0.04) in experimental data. In TCGA samples, alpha-fetoprotein levels (mg/dL) were negatively correlated with the expression of SEP15 (p<0.001), SELENOP (p<0.001), SOD1 (p<0.001), SOD2 (p<0.001), CAT (p<0.001), and NFE2L2 (p=0.004). Alpha-fetoprotein levels were positively correlated with the expression of GPX4 (p=0.02) and SELENOP (p=0.01) in the experimental data. Low expression of GPX1 (p=0.006), GPX4 (p=0.01), SELENOP (p=0.006), SOD1 (p=0.007), CAT (p<0.001), and NFE2L2 (p<0.001), and higher levels of GSR, were associated with low overall survival at 12 months. These results suggest a significant role for these antioxidant enzymes in HCC pathogenesis and severity.

## Introduction

Hepatocellular carcinoma (HCC) has a high mortality rate, and ranks as the third leading cause of cancer deaths worldwide (Ozakyol, 2017; Forner et al., 2018). Major risk factors for HCC include infection with hepatitis B and C viruses, alcohol intake, and fatty liver disease (Ozakyol, 2017; Yang et al., 2019). Prognosis and treatment options vary according to tumor stage and liver function. The percentage of patients eligible for curative treatment varies between high and low-resource countries (Ozakyol, 2017; Yang *et al.,* 2019), but generally fluctuates between 20-30% of patients. The median survival of patients with untreated disease is nine months (Klungboonkrong et al., 2017; Forner *et al.,* 2018). New markers or therapeutic targets are required for early diagnosis, and the development of novel treatment strategies for HCC (Klungboonkrong *et al.,* 2017).

Oxidative stress is associated with cancer, and has a dual role in disease development, due to the effects of reactive oxygen species (ROS) on cellular processes. Increased ROS levels are associated with oncogenic effects because of their ability to cause damage to biological macromolecules, such as DNA, lipids, and proteins (Reuter et al., 2010; Moloney and Cotter, 2018). High concentrations of ROS have been associated with activation of p53, oxidative lipid peroxidation, consumption of antioxidants, and can ultimately lead to cell death (Wang et al., 2016b; Sajadimajd and Khazaei, 2017). Oxidative stress can play different roles: promoting carcinogenesis or cell apoptosis, or by providing sufficient components to promote cancer cell survival. The physiological functions of ROS include regulation of the expression and activity of several signaling regulators that are involved in key processes, such as proliferation and apoptosis (Gill et al., 2016). Cancer cells, in turn, seem to maintain advantageously elevated levels of ROS to guarantee their survival, by adapting the content and regulation of their antioxidant machinery (Sajadimajd and Khazaei, 2017; Moloney and Cotter, 2018).

Cellular antioxidant defense systems include a series of antioxidant enzymes that maintain homeostasis by restricting ROS production or neutralizing ROS (Gill et al., 2016). This group of enzymes includes major components such as superoxide dismutase (SOD), catalase (CAT), and glutathione reductase (GSR), and selenoproteins, including glutathione peroxidase 1 (GPX1), glutathione peroxidase 4 (GPX4), 15-kDa selenoprotein (SEP15), and selenoprotein P (SelP). All of these enzymes act in pathways of chain-breaking ROS molecules, or have other important functions, including detoxification of hydrogen peroxide (H_2_O_2_), inhibition of lipid peroxidation, quality control of protein folding, and transport of selenium to peripheral tissues (Gupta et al., 2014; Labunskyy et al., 2014; Zoidis et al., 2018). Altered expression of these enzymes could be a useful resource for cancer cells. Hyperactivation of nuclear factor erythroid 2-related factor 2 (Nrf2), a transcription factor that regulates the expression of several genes, including antioxidant enzymes, has been associated with a variety of cancers as well as with HCC (Cheng et al., 2015; Menegon et al., 2016; Ma-on et al., 2017; Sajadimajd and Khazaei, 2017).

Previous studies have demonstrated an association between aberrant expression of antioxidant enzymes and cancer (Table 1). Abnormal expression of the *GPX1, GPX4, SEP15*, and *selenoprotein P* (*SELENOP*) genes has been detected in a variety of cancers, including gastric cancer (Lan et al., 2017), colon carcinoma (Yagublu et al., 2011), colorectal cancer (Hughes et al., 2018), clear cell renal cell carcinomas (Rudenko et al., 2015; Cheng et al., 2019), laryngeal squamous cell carcinoma (Zhang Q et al., 2018), breast cancer (Król et al., 2018), MCF-7 adenocarcinoma cells (Rusolo et al., 2017), lung cancer (Gresner et al., 2009), and HepG2 liver cancer cells (Guariniello et al., 2015; Zhao et al., 2015). The *SOD1, SOD2, glutathione-disulfide reductase* (*GSR*), *catalase* (*CAT*)*,* and *nuclear factor erythroid 2-related factor 2* (*NFE2L2*) genes are deregulated in bladder cancer (Wieczorek et al., 2017), oral squamous cell carcinoma (Pedro et al., 2018), breast cancer (Wolf et al., 2016), lung cancer (Zhang Y *et al.,* 2016), MCF-7 cells (Shi et al., 2017), and HCC (Cheng *et al.,* 2015; Guerriero et al., 2015; Wang et al., 2016a).

**Table 1 -:** Comparison of gene expression in the present study and literature.

Type of cancer	GPX1	GPX4	SEP15	SELENOP	SOD1	SOD2	GSR	CAT	NFE2L2
Colon carcinoma (Yagublu *et al*., 2011)	↑	↑	-	-	-	-	-	-	-
Colorectal cancer (Hughes *et al*., 2018)	↑	NS	-	↓	-	↑	-	-	-
Bladder cancer (Wieczorek *et al*., 2017)	↑	-	-	-	-	NS	-	NS	-
Laryngeal squamous cell carcinoma (Zhang *et al*., 2018)	↑	-	-	-	-	-	-	-	-
Gastric Cancer (Lan *et al*., 2017)	↓	↓	↓	-	-	-	-	-	-
Clear cell renal cell carcinomas (Rudenko *et al*., 2015)	↓	↓	-	-	-	-	-	-	-
Clear cell renal cell carcinomas (Cheng *et al*., 2019)	↑	NS	-	-	-	-	-	-	-
Breast Cancer (Król *et al*., 2018)	↓	-	-	-	-	-	-	-	-
Breast Cancer (Wolf *et al*., 2016)	-	-	-	-	-	-	-	-	↓
MCF 7 cells (Rusolo *et al*., 2017)	↓	↓	-	-	-	-	-	-	-
MCF 7 cells (Shi *et al*., 2017)	-	-	-	-	-	↑	-	-	-
Non-small cell lung cancer (Gresner *et al*., 2009)	-	-	NS	↓	-	-	-	-	-
Lung squamous cell carcinoma (Zhang *et al*., 2016)	-	-	-	-	-	-	-	-	↑
Oral squamous cell carcinoma (Pedro *et al*., 2018)	-	-	-	-	↓	↑	↓	↓	-
Hepatocellular carcinoma (Wang *et al*., 2016)	-	-	-	-	-	↓	-	-	-
Hepatocellular carcinoma (Guerriero *et al*., 2015)	-	↑	-	-	-	-	-	-	-
Hepatocellular carcinoma (Cheng *et al*., 2015)	-	-	-	-	-	-	-	-	↑
HepG2 cell line (Zhao *et al*., 2015)	↑	↑	-	↑	-	-	-	-	-
HepG2 and Huh7 cell lines (Guariniello *et al*., 2015)	-	↑	↑	-	-	-	-	-	-
TCGA (Tumor x normal adjacent tissue)	↑	NS	NS	↓	↓	↓	NS	↓	↓
TCGA x GTEx (Case x control)	↑	↑	↑	↑	NS	↓	↑	↓	↑
ISCMPA (Tumor x peritumor)	NS	↑	NS	NS	NS	↑	NS	NS	NS

Abbreviations: ↑=higher expression in tumoral compared to normal/peritumoral tissue (or non-tumoral cell line); ↓= lower expression in tumoral compared to normal/peritumoral tissue (or non-tumoral cell line); NS = not significant; - = not analyzed; TCGA= The Cancer Genome Atlas; GTEx = Genotype-Tissue Expression; ISCMPA = Irmandade Santa Casa de Misericórdia de Porto Alegre. Each line corresponds to one study for better understanding. MCF 7 cells constitute a type of breast cancer cell line, and HepG2 and Huh7 cells constitute types of HCC and liver cancer cell line.

In the present study, we produced new data on the gene expression levels of the antioxidant enzyme genes *GPX1, GPX4, SEP15, SELENOP, SOD1, SOD2, GSR*, *CAT,* and *NFE2L2* in human HCC tissues. Bioinformatic analyses using databases and RT-qPCR analysis of the original data were performed to investigate whether changes in the expression of these genes might be associated with severity and overall survival in HCC, and to explore possible relationships between the genes.

## Material and Methods

### Datasets and bioinformatic analyses of differentially expressed genes

Bioinformatic analyses were performed using two different experimental designs: transversal and case-control studies. For the transversal study, publicly available RNA-Seq data from the liver hepatocellular carcinoma (LIHC) project were downloaded directly from The Cancer Genome Atlas (TCGA) portal. These data included HTSeq-Counts of matched samples from 48 tumoral tissues and 48 normal solid tissues. The results published here are in whole or part based upon data generated by the TCGA Research Network: https://www.cancer.gov/tcga. For the case-control study, RNA-Seq by Expectation-Maximization expected count data of 292 TCGA-LIHC tumoral samples (case) and 115 Genotype-Tissue Expression (GTEx) normal liver samples (control) were downloaded from “*Figshare Data Record 1”*, made available by Wang et al. (2018). Since TCGA and GTEx are studies from different sources, reprocessing of data and batch effect removal were necessary for adequate comparison. Therefore, in the present study, normalized datasets provided by Wang *et al.* (2018) were used. Publicly available clinical data were also collected from both datasets.

Differential expression analyses for both studies (tumoral × normal and case × control) were performed using the DESeq2 (Love et al., 2014) package in the R language with padj<0.05. The Edge R (Robinson et al., 2009) package was also used for trimmed mean of M values (TMM) normalization and generation of logarithmic counts per million (logCPM) data for further statistical analyses.

### Tissue samples and clinical data collection

Hepatic fresh tissue specimens (tumoral and adjacent peritumoral tissues) were collected from 14 cirrhotic patients with HCC who underwent liver transplantation between 2013 and 2015 at the Division of Gastroenterology of Irmandade Santa Casa de Misericórdia de Porto Alegre (ISCMPA), Brazil. Clinical data, such as age, sex, etiology, and metabolic panel, were collected from medical records. Informed consent was obtained from all patients. The study protocol was approved by the ISCMPA and Universidade Federal de Ciências da Saúde de Porto Alegre (UFCSPA) Ethics Committees (no. 2.400.119).

### RNA extraction and quantitative real-time PCR analysis

Tissue samples from ISCMPA were collected from explanted liver and immediately dipped in RNAlater solution Ambion® (Thermo Fisher Scientific, USA). The stabilized tissue samples were frozen at −80 °C until RNA isolation. Total RNA was extracted from the samples shortly after collection, using TRIzol™ reagent (Invitrogen, USA) according to the manufacturer’s specifications. RNA purity and concentration were evaluated by spectrometry using a Biospec-Nano device (Shimadzu, Japan). RNA integrity was evaluated by agarose gel electrophoresis of total RNA (Figure S1). Total RNA was reverse transcribed using the GoScript™ Reverse Transcription System (Promega, USA) according to the manufacturer’s instructions, in a PCR thermal cycler (Applied Biosystems, USA). Total RNA not used for RT-PCR was frozen at −80 °C in case any additional experiments were necessary.

Quantitative polymerase chain reaction (qPCR) assays were performed on a StepOnePlus™ system (Applied Biosystems, USA), using SYBR™ Select Master Mix (Applied Biosystems, USA) and specific primers (Invitrogen, USA). The primer sequences are shown in Table S1. *Actin beta* (*ACTB*)*, glyceraldehyde 3-phosphate dehydrogenase* (*GAPDH*)*,* and *18S* genes were tested for stability using the protocol described by Moura et al. (2014). The *ACTB* gene displayed higher stability, and was used as an endogenous control.

Gene expression was normalized to the *ACTB* housekeeping gene. The difference in gene expression between tumoral and peritumoral tissues (used as the calibrator) was calculated using the 2^−ΔΔCt^ method (Livak and Schmittgen, 2001; Schmittgen and Livak, 2008), where ΔΔCt= ΔCt(tumor)-ΔCt(peritumor) for tumoral tissue and ΔΔCt=ΔCt(peritumor)-ΔCt(peritumor) for peritumoral tissue. Fold-change calculations were conducted as previously described (Schmittgen and Livak, 2008).

### Protein-protein interaction (PPI) network analysis

PPI network visualization and analysis were performed using Cytoscape 3.8 software (Shannon, 2003). PPI network construction was carried out using the Search Tool for the Retrieval of Interacting Genes (STRING) database v.11 (Szklarczyk et al., 2019), using STRINGapp from Cytoscape 3.8. A confidence score of 0.4 was set as the cut-off criterion.

### Gene ontology (GO) and pathway enrichment analyses

To analyze the genes and biological characteristics, bioinformatic enrichment analysis of GO and pathways was performed using the STRINGapp plugin from Cytoscape 3.8. This plugin has a feature that performs enrichment retrieval from databases. GO (Ashburner et al., 2000; Carbon et al., 2019), the Kyoto Encyclopedia of Genes and Genomes (KEGG) Pathway (Kanehisa, 2000; Kanehisa *et al.,* 2019) and REACTOME Pathway (Jassal et al., 2020) were the databases selected for retrieval by the STRINGapp plugin. Two groups of genes were examined separately: upregulated and downregulated. All genes were analyzed with “*Homo sapiens*” as background species. The statistical criterion was a corrected p-value <0.05.

### Statistical analyses

Continuous data are shown as mean ± standard deviation or median (interquartile range). Categorical values are shown as absolute frequency (relative frequency). Shapiro-Wilks or Kolmogorov-Smirnov tests were used to test the normality of variables. Comparison of mRNA levels between tumoral and peritumoral tissues was performed using Wilcoxon signed-rank tests. Correlations were made using Spearman’s tests, and are presented as p-values and r coefficients. Survival analysis was performed using Kaplan-Meier log-rank tests and Cox regression. Evaluate Cutpoints (Ogłuszka et al., 2019) software was used to choose the optimal cut-off point for the dichotomization of continuous variables. SPSS 20.0 (SPSS Inc., USA) and R software version 4.0.0 were used for statistical analysis. The significance level was set at p<0.05.

## Results

### Differential gene expression of antioxidant enzymes in HCC

**Figure 1 -:**
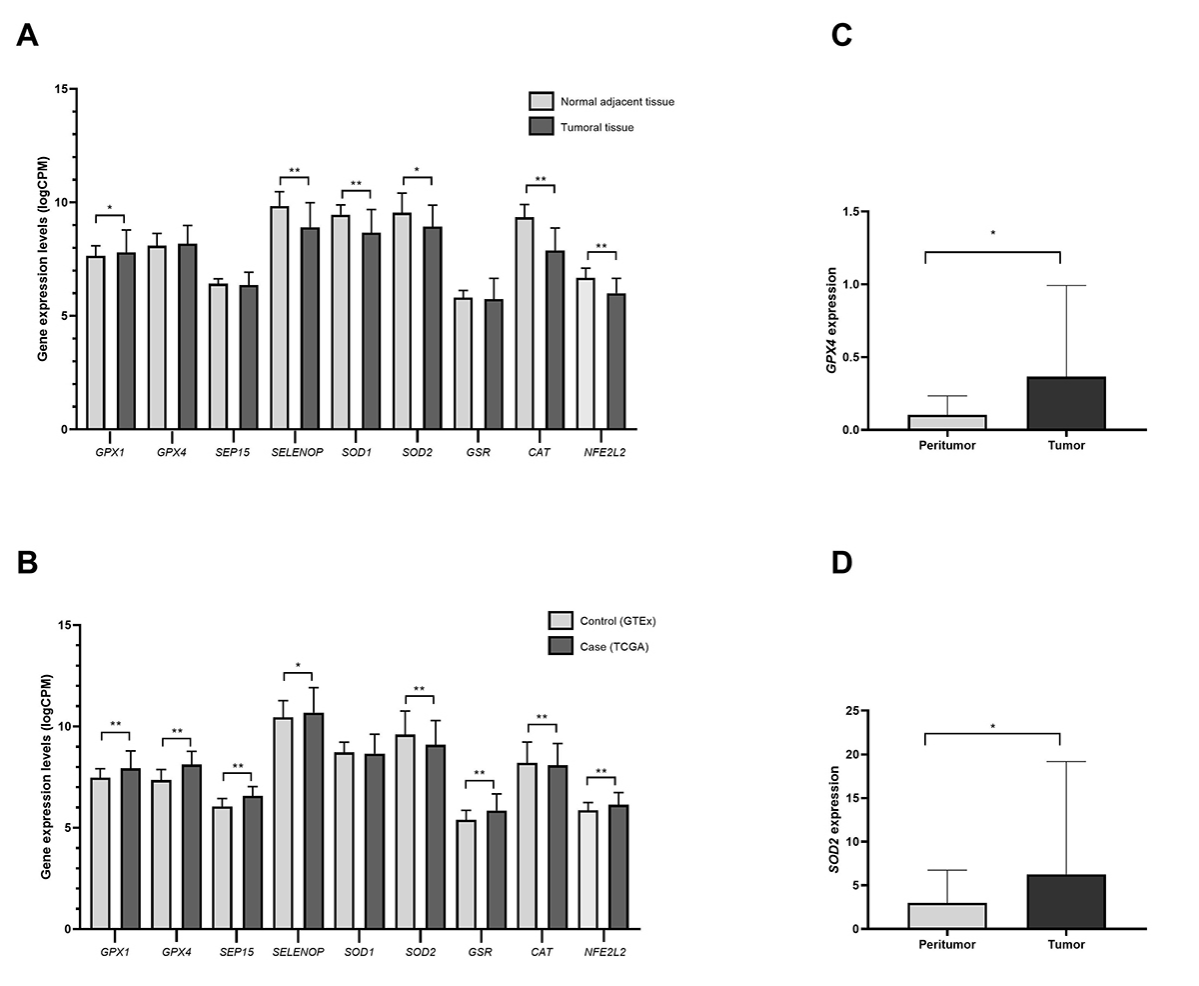
Gene expression profiles. (A) Gene expression levels in the TCGA-LIHC dataset for tumoral and normal adjacent tissues. (B) Gene expression levels in case (TCGA) versus control (GTEx) analysis. (C) and (D) Significant differentially expressed genes in ISCMPA’s sample (tumoral versus peritumoral tissues). *p<0.05, **p<0.001.

Comparison of matched tumoral and adjacent normal tissues from TCGA patients revealed significantly differential expression of six genes (Figure 1A). One gene (*GPX1*) was upregulated and five *(SELENOP, SOD1, SOD2, CAT*, and *NFE2L2*) were downregulated. All genes except *SOD1* showed significant differential expression in TCGA (case) and GTEx (control) comparisons (Figure 1B). Six genes (*GPX1, GPX4, SEP15, SELENOP, GSR, and NFE2L2*) were upregulated, and two genes (*SOD2* and *CAT*) were downregulated. Detailed data from these analyses are provided in Table S2.

In RT-qPCR analysis of 28 fresh frozen matched samples of HCC patients from ISCMPA, two genes displayed significantly higher expression in tumoral tissue than in peritumoral tissue (Figure 1C, D). *GPX4* displayed a 2.70-fold increase (p=0.02), and *SOD2* a 2.59-fold increase (p=0.04). In contrast, the *GPX1, SEP15, SELENOP, SOD1*, *GSR, CAT,* and *NFE2L2* genes were not significantly differentially expressed when comparing tumoral and peritumoral tissues (data not shown).

### Correlation analysis

**Figure 2 -:**
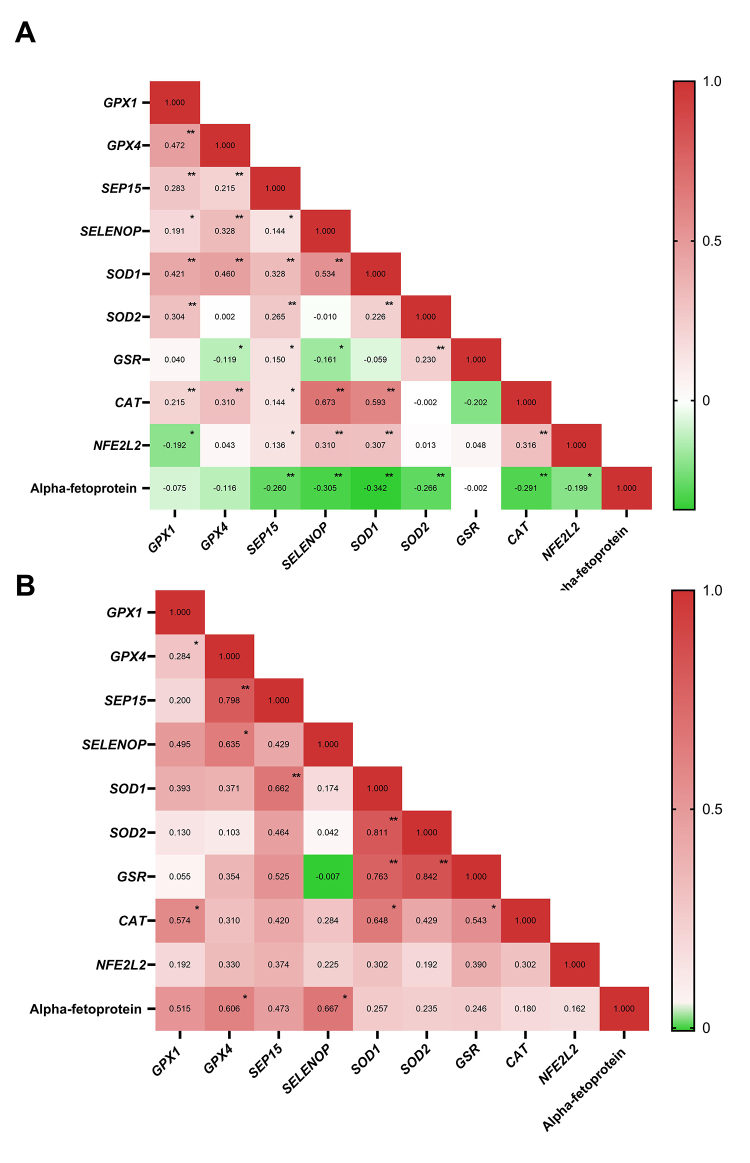
Spearman correlation analysis. (A) Spearman correlation coefficients (r) for gene expression levels in TCGA tumoral samples and clinical data. (B) Spearman correlation coefficients (r) for gene expression levels in ISCMPA tumoral samples. *p<0.05, **p<0.001.

Correlations between gene expression levels of TCGA data and alpha-fetoprotein levels were also examined (Figure 2A). Alpha-fetoprotein levels (mg/dL) were negatively correlated with the expression of *SEP15* (p<0.001), *SELENOP* (p<0.001), *SOD1* (p<0.001), *SOD2* (p<0.001), *CAT* (p<0.001), and *NFE2L2* (p=0.004). In contrast, analysis of relative mRNA expression (2^−ΔΔCt^) in tumoral tissue revealed that patients from ISCMPA, alpha-fetoprotein (mg/dL) levels were positively correlated with *GPX4* (p=0.02) and *SELENOP* (p=0.01) expression levels (Figure 2B).

### Survival analysis

**Figure 3 -:**
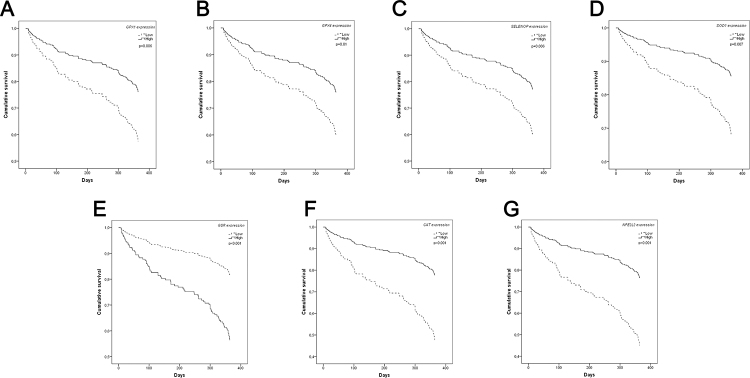
Survival analysis. Cumulative survival plot of dichotomized gene expression levels of TCGA tumoral tissues.

In TCGA samples, patients were divided into two groups according to gene expression (high or low), using the optimal cut-off point generated by the Evaluate Cutpoints (Ogłuszka et al., 2019) software. Kaplan-Meier and Cox multivariate regression were used to generate overall survival data (Figure 3 and Table 2). The final models used for multivariate analysis are presented in Table S3. Low overall survival at 12 months was correlated with low expression of *GPX1* (p=0.006), *GPX4* (p=0.01), *SELENOP* (p=0.006), *SOD1* (p=0.007), *CAT* (p<0.001), and *NFE2L2* (p<0.001). Higher levels of *GSR* (p<0.001) were associated with low overall survival in the same period. Complete sample data from patients from ISCMPA were not available for survival analysis.

**Table 2 -:** Multivariate Cox proportional hazards regression analysis to assess the independent effect of gene expression on survival in 12 months

	Adjusted HR	95% CI	p
*GPX1*	2.023	1.222-3.347	0.006
*GPX4*	1.859	1.147-3.015	0.012
*SEP15*	0.75	0.315-1.783	0.514
*SELENOP*	1.968	1.217-3.183	0.006
*SOD1*	2.447	1.273-4.702	0.007
*SOD2*	1.461	0.824-2.590	0.194
*GSR*	0.354	0.224-0.559	<0.001
*CAT*	2.924	1.774-4.820	<0.001
*NFE2L2*	2.955	1.779-4.908	<0.001

HR: hazard ratio; CI: confidence interval

### PPI network analysis

**Figure 4 -:**
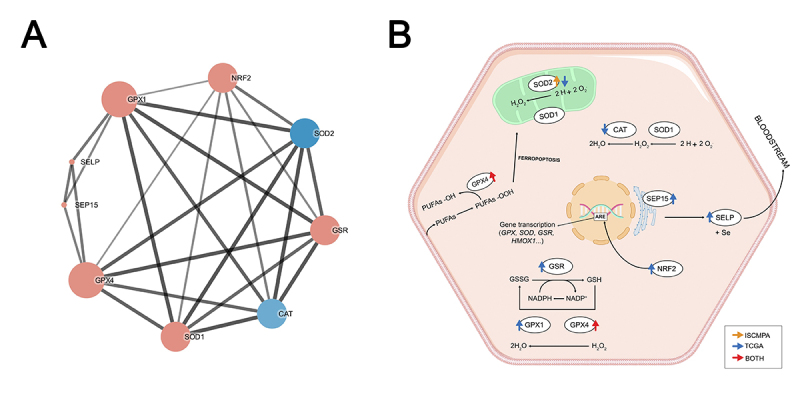
Enriched annotation pathways and interaction network analysis of antioxidant proteins investigated in the present study. (A) Protein-protein interaction network of the analyzed genes. Lines represent interaction associations between nodes and line thickness indicates the strength of data support (score). Size of nodes indicates the degree of associations. Blue nodes represent downregulated genes and red nodes represent upregulated genes according to data from case (TCGA) vs control (GTEx) analyses. (B) Illustrative representation of antioxidant enzymes functions in hepatocytes. Up- and down-arrows denote significant upregulated or downregulated genes, respectively. Red arrows: both analysis; Blue arrows: only in TCGA analysis; Orange arrows: only in experimental analysis. Abbreviations: GPX1, glutathione peroxidase 1; GPX4, glutathione peroxidase 4; GSR, glutathione reductase; SOD1, superoxide dismutase 1; SOD2, superoxide dismutase 2; CAT, catalase; SEP15, 15 KDa selenoprotein; SelP, selenoprotein P; GSSG, oxidized glutathione; GSH, reduced glutathione; NRF2; nuclear factor erythroid 2-related factor 2; HMXO1; heme oxygenase-1 gene; ARE; antioxidant response element; Se, selenium; and PUFAs; Polyunsaturated fatty acids.

Since correlation analyses revealed several significant correlations (Figure 2) between gene expression in both TCGA and ISCMPA samples, a PPI network analysis was performed. The PPI network contained nine nodes and 25 edges (Figure 4A), with an average node degree of 5.55 and PPI enrichment p-value < 1.0e-16. GPX1 and GPX4 had the highest degree and betweenness centrality values, of 8 and 0.166, respectively. Proteins SELP and SEP15 had the lowest degree value, interacting with only three other proteins in this analysis: GPX1, GPX4, and each other.

### GO and pathway enrichment analyses

Gene enrichment analysis using the enrichment tool STRINGapp returned a series of sets. GO terms are divided into three groups: biological process (BP), molecular function (MF), and cellular component (CC). The most significant results are presented in Table 3.

**Table 3 -:** Top Most significant enriched gene ontology (GO) terms.

Category	Id	Term	Count	Genes	P-value^a^
Upregulated					
BP	GO.0098754	Detoxification	5	*GSR|SOD1|GPX4|NFE2L2|GPX1*	5.92E-08
BP	GO.0006979	Response to oxidative stress	6	*GSR|SOD1|GPX4|NFE2L2|GPX1|SELENOP*	1.50E-07
BP	GO.0097237	Cellular response to toxic substance	5	*GSR|SOD1|GPX4|NFE2L2|GPX1*	6.26E-07
BP	GO.0098869	Cellular oxidant detoxification	4	*GSR|SOD1|GPX4|GPX1*	3.10E-06
BP	GO.1902175	Regulation of oxidative stress-induced intrinsic apoptotic signaling pathway	3	*SOD1|NFE2L2|GPX1*	2.18E-05
BP	GO.0034599	Cellular response to oxidative stress	4	*GSR|SOD1|NFE2L2|GPX1*	6.32E-05
BP	GO.0006749	Glutathione metabolic process	3	*GSR|SOD1|GPX1*	6.95E-05
BP	GO.0045454	Cell redox homeostasis	3	*GSR|NFE2L2|GPX1*	1.20E-04
BP	GO.0042542	Response to hydrogen peroxide	3	*SOD1|NFE2L2|GPX1*	4.20E-04
BP	GO.0019372	Lipoxygenase pathway	2	*GPX4|GPX1*	7.50E-04
MF	GO.0016209	Antioxidant activity	4	*GSR|SOD1|GPX4|GPX1*	7.40E-07
MF	GO.0004602	Glutathione peroxidase activity	2	*GPX4|GPX1*	8.10E-04
MF	GO.0016491	Oxidoreductase activity	4	*GSR|SOD1|GPX4|GPX1*	0.0014
CC	GO.0070013	Intracellular organelle lumen	7	*GSR|SOD1|SEP15|GPX4|NFE2L2|GPX1|SEPP1*	0.0067
CC	GO.0005759	Mitochondrial matrix	3	*GSR|SOD1|GPX1*	0.0082
CC	GO.0005739	Mitochondrion	4	*GSR|SOD1|GPX4|GPX1*	0.0163
Downregulated					
BP	GO.0000302	Response to reactive oxygen species	2	*CAT|SOD2*	0.0057
BP	GO.0034599	Cellular response to oxidative stress	2	*CAT|SOD2*	0.0057
BP	GO.0051289	Protein homotetramerization	2	*CAT|SOD2*	0.0057
BP	GO.0072593	Reactive oxygen species metabolic process	2	*CAT|SOD2*	0.0057
BP	GO.0098869	Cellular oxidant detoxification	2	*CAT|SOD2*	0.0057
BP	GO.0007568	Aging	2	*CAT|SOD2*	0.006
BP	GO.0010035	Response to inorganic substance	2	*CAT|SOD2*	0.0157
BP	GO.0043066	Negative regulation of apoptotic process	2	*CAT|SOD2*	0.0419
BP	GO.0055114	Oxidation-reduction process	2	*CAT|SOD2*	0.043
MF	GO.0016209	Antioxidant activity	2	*CAT|SOD2*	5.8E-4
MF	GO.0016491	Oxidoreductase activity	2	*CAT|SOD2*	0.0229

GO, gene ontology; BP, biological process; MF, molecular function; CC, cellular component; ^a^ Corrected p-value

Upregulated genes were mainly enriched in the biological processes of cellular detoxification, response to oxidative stress, and cellular response to toxic substances. With respect to GO molecular function, antioxidant activity and glutathione peroxidase activity were implicated. According to GO cellular component, genes was mainly enriched in the intracellular organelle lumen. Downregulated genes were mainly enriched in biological processes of response to ROS, protein homotetramerization, and aging. GO molecular functions returned antioxidant activity and oxidoreductase activity as the main enriched terms.

KEGG pathways and REACTOME Pathways analyses revealed that genes were enriched in several pathways. The most significant results are presented in Table 4. Upregulated genes were mainly enriched in pathways involving glutathione metabolism, synthesis of eicosatetraenoic acids, detoxification of ROS, and diseases that included amyotrophic lateral sclerosis and Huntington’s disease. Downregulated genes were also enriched in pathways of detoxification of ROS, as well as pathways involving peroxisomes, longevity regulation, FoxO signaling, and the immune system.

**Table 4 -:** Top Most significant enriched pathways.

Category	Id	Term	Count	Genes	P-value^a^
Upregulated					
K	hsa00480	Glutathione metabolism	3	GSR|GPX4|GPX1	8.46E-06
R	HSA-2142770	Synthesis of 15-eicosatetraenoic acid derivatives	2	GPX4|GPX1	7.05E-05
R	HSA-2142712	Synthesis of 12-eicosatetraenoic acid derivatives	2	GPX4|GPX1	7.05E-05
R	HSA-2142688	Synthesis of 5-eicosatetraenoic acids	2	GPX4|GPX1	7.05E-05
R	HSA-3299685	Detoxification of Reactive Oxygen Species	2	SOD1|GPX1	4.20E-04
K	hsa05014	Amyotrophic lateral sclerosis (ALS)	2	SOD1|GPX1	9.40E-04
K	hsa04918	Thyroid hormone synthesis	2	GSR|GPX1	0.0013
R	HSA-114608	Platelet degranulation	2	SOD1|SEPP1	0.0033
K	hsa05016	Huntington’s disease	2	SOD1|GPX1	0.0065
Downregulated					
R	HSA-3299685	Detoxification of Reactive Oxygen Species	2	CAT|SOD2	6.61E-5
K	hsa04146	Peroxisome	2	CAT|SOD2	9.18E-5
K	hsa04211	Longevity regulating pathway	2	CAT|SOD2	9.18E-5
K	hsa04068	FoxO signaling pathway	2	CAT|SOD2	1.0E-4
R	HSA-2262752	Cellular responses to stress	2	CAT|SOD2	0.003
R	HSA-8953897	Cellular responses to external stimuli	2	CAT|SOD2	0.003
R	HSA-168256	Immune System	2	CAT|SOD2	0.046

K, KEGG Pathways; R, REACTOME Pathways; a Corrected p-value

## Discussion

We performed bioinformatic and experimental analyses to evaluate the expression patterns of eight antioxidant enzymes, including four selenoproteins, and one important transcription factor, to assess their association with HCC pathogenesis. A number of differentially expressed genes were identified in tumoral samples from TCGA patients (Figure 1A, B), along with replication of two genes in the experimental data (Figure 1C, D). Previous studies have demonstrated variable expression patterns of antioxidant genes, depending on the type of cancer analyzed (Table 1). The present results add new information about the expression of these genes in HCC.

Some studies evaluated the expression of the genes for these antioxidant enzymes separately in HCC tumoral samples, HepG2 and Huh7 cell lines (Table 1). Results for *GPX1, GPX4, SEP15, SELENOP, SOD2*, and *NFE2L2* seem to be corroborated by our analysis (Cheng et al., 2015; Guariniello et al., 2015; Guerriero et al., 2015; Wang et al., 2016a; Zhao et al., 2015). We observed that the pattern of gene expression of these enzymes seemed to vary when analysis was performed in TCGA tumoral versus normal adjacent tissues, and TCGA versus GTEx databases (Figure 1A, B and Table 1). The tumor microenvironment seems to play a role in HCC progression, influencing progression by modulation of liver fibrosis, initiation of the epithelial-to-mesenchymal transition, invasion, alterations of oxidative stress status, and other processes (Novikova et al., 2017). Differences in antioxidant enzyme levels between normal, tumoral, and peritumoral tissues may represent different stages of adaptation of this system against oxidative stress, or the use of ROS as signaling molecules (Moloney and Cotter, 2018).

Differences in the expression of two genes, *GPX4* and *SOD2*, were statistically significant in TCGA and experimental data. Increased expression of *GPX4* was present in TCGA versus GTEx analysis and experimental data (Figure 1B, C). However, this difference was not significant in TCGA tumoral × non-tumoral tissues (Figure 1A). In agreement with our findings, studies with grade III HCC tissues (Guerriero et al., 2015), HepG2 cell lines (Guariniello et al., 2015; Zhao et al., 2015), and Huh7 cells (Guariniello *et al.,* 2015) also revealed higher expression of *GPX4* (Table 1). Lower expression was found in gastric cancer (Lan et al., 2017), clear cell renal cell carcinoma (Rudenko et al., 2015), and human breast cancer cell lines (Rusolo et al., 2017) (Table 1).

GPX4 has the same detoxification function as GPX1 in the cell, including the ability to reduce lipid peroxides (Figure 4B) (Labunskyy et al., 2014). Even though mRNA expression was analyzed, and gene transcription is not always directly related to protein synthesis, it is possible that overexpression of *GPX4* could affect the cell environment. Increased *GPX4* expression could enhance the levels of antioxidant components in cells and protect against oxidative stress (Davis et al., 2012; Rohr-Udilova et al., 2018). However, overexpression of GPX4 could also benefit cancer cells. GPX4 plays an important role in preventing oxidative stress-induced apoptosis by decreasing lipid peroxidation. In so doing, GPX4 blocks posterior signaling, leading to cell death (Figure 4B). Therefore, overexpression of this enzyme could be an advantageous mechanism used by tumoral cells to sustain growth and avoid apoptosis (Labunskyy *et al.,* 2014; Rohr-Udilova *et al.,* 2018). A previous study showed that overexpression of GPx4 in HCC *in vitro* protected the cells from oxidative stress and reduced the intracellular free radical level (Rohr-Udilova *et al.,* 2018).

*SOD2* had different expression patterns between TCGA analyses and experimental data (Figure 1A, B, D and Table 1). Reduced expression of *SOD2* has been previously reported (Wang et al., 2016a), in a study into HBV‐positive HCC tumors in a cohort. Higher gene expression of *SOD2* has been reported in oral squamous cell carcinoma (Pedro et al., 2018) and colorectal adenoma and cancer (Hughes et al., 2018). Most of the patients from TCGA and Wang *et al.* (2016a) presented with hepatitis B as the main etiology, while patients from our experimental data were mostly HCV-positive. No statistically significant differences were found in gene expression levels between risk factor types (data not shown). A diversity of tumor types can develop in HCC, in terms of staging and its molecular subclasses, which could explain, in part, the variety of findings in gene expression and deregulated pathways (Hoshida et al., 2013).

SOD2 is located in the mitochondrial matrix (Figure 4B) and acts to catalyze dismutation of the superoxide anion (O_2_^•−^) to H_2_O_2_, playing a crucial role in alleviating oxidative stress (Kim et al., 2017). Loss of this antioxidant component could impair the oxidative balance in cells. However, its overexpression could favor the cancer cell environment (Kim *et al.,* 2017). Intensification of SOD2 expression in tumoral cells seems to ensure H_2_O_2_ flow from mitochondria, which is a crucial step for the occurrence of the Warburg effect (Che et al., 2016), a strategy used by cancer cells to increase the generation of additional metabolites. Upregulation of *SOD2* could favor H_2_O_2_ accumulation (Figure 4B), which is involved in a variety of signaling pathways related to proliferation, migration, and invasion in cancer cells (Glorieux et al., 2015).

Survival analysis of patients from TCGA revealed an interesting aspect of gene expression patterns in HCC. Although higher expression of antioxidant enzymes was present in HCC patients, patients with lower gene expression also displayed lower overall survival, except for *GSR* expression (Figure 3). As previously discussed, ROS act as a two-edged sword in cancer, with beneficial and detrimental roles in cells, and are tightly regulated by cancer cells (Moloney and Cotter, 2018). On the one hand, higher expression of antioxidant enzymes could not only control ROS accumulation, preventing cell death being triggered by them, but could also perpetuate tumoral cells. On the other hand, lower expression could cause apoptosis and kill tumoral cells. However, regarding the level of expression, tumors could also be favored due to the accumulation of ROS used as signaling factors (Sajadimajd and Khazaei, 2017; Moloney and Cotter, 2018). This mechanism and the extent to which the levels of these antioxidant enzymes vary in each stage of hepatocarcinogenesis should be explored in depth in further studies.

Correlation (Figure 2), PPI network (Figure 4A), and GO (Tables 3 and 4) analyses highlighted possible relationships between the antioxidant enzymes investigated and their interactions in different pathways (Table 4). NFR2, which is encoded by *NFE2L2*, seems to be important because of its interaction with Antioxidant Responsive Element (ARE) (Figure 4B), an interaction that increases the expression of several genes, including genes that encode glutathione peroxidases and SODs (Raghunath et al., 2018). Further analysis at the protein level should be performed to clarify the knowledge about this protein network, especially in the context of carcinogenesis.

None of the other genes in the experimental data had significantly altered expression, even though there was a tendency towards underexpression or overexpression of some genes. The limitations of our study, such as the difficulty in acquiring fresh liver tissue for mRNA analysis, resulted in a small sample size, which could be one of the explanations for the lack of statistical significance. To improve our analysis, we also investigated data from larger databases, such as TCGA and GTEx, which provided us with a better understanding of the results. In addition, peritumoral tissue, due to its proximity to the tumor, could already contain alterations, and could complicate the examination of gene expression differences. However, the availability of fresh, healthy liver tissue was also limited. Peritumoral tissue was collected with a safety margin and, in this case, allowed us to perform a paired analysis. These data permitted an evaluation of differences in gene expression in the tumoral and adjacent peritumoral tissues of the same patient. To verify the presence of differences between tissue types, we performed bioinformatic analyses between matched samples, as well as between case and control samples.

We present preliminary findings of the gene expression patterns of antioxidant enzymes in HCC. The findings highlight the importance of further evaluation of these components in the pathology of cancer studies with larger sample sizes. We were able to replicate data from TCGA analysis for at least two genes. The present study is one of few investigations to investigate a diversity of antioxidant enzyme genes in the context of cancer. We were also able to examine clinical parameters and survival data for different gene expression levels in TCGA patients. Our study highlights the need for further studies to better understand the role of these enzymes in HCC.

## Supplementary material

The following online material is available for this article:

Table S1 - Sequences of primer pairs.

Table S2 - Detailed results of gene expression analysis using RNA-Seq data.

Table S3 - Final models of multivariate Cox regression analysis.

Figure S1 - Sample of agarose gel electrophoresis of total RNA.
