# Supplementary material for: Gene expression evaluation of antioxidant enzymes in patients with hepatocellular carcinoma: RT-qPCR and bioinformatic analyses
Source: Genet Mol Biol. 2021 Apr 2;44(2):e20190373. doi: 10.1590/1678-4685-GMB-2019-0373 (PMC8022359; doi:10.1590/1678-4685-GMB-2019-0373)
Supplement: Table S1 - [file 1415-4757-GMB-44-2-e20190373-s1.pdf]

Genetics and Molecular Biology
https://doi.org/10.1590/1678-4685-GMB-2019-0373

Supplementary Material to "Gene expression evaluation of antioxidant enzymes in patients with hepatocellular carcinoma: RT-qPCR and bioinformatic analyses"

Table S1 - Sequence of primers pairs.

Gene	Gene ID	Sequence
ACTB	60	F: 5' TGACGTGGACATCCGCAAAG 3'<br>R: 5' CTGGAAGGTGGACAGCGAGG3'
GPX1	2876	F: 5' AACCAGTTTGGGCATCAGGAGA 3'<br>R: 5' TCTCGAAGAGCATGAAGTTGGG 3'
GPX4	2879	F: 5' TGTAACCAGTTCGGGAAGCA 3'<br>R: 5' TCCACTTGATGGCATTTCCC3'
SEP15	9403	F: 5' GTCCAAGCTTTTGTTAGGAGTG 3'<br>R: 5' CAGCAATGTTCCCATTGTCGT 3'
SELENOP	6414	F: 5' AAACTGCTCTCTCACGACTCT 3'<br>R: 5' GATGGTAATGAGGCGATGGAGT 3'
SOD1	6647	F: 5' GAGTTTGGAGATAATACAGCAGGCTGT 3'<br>R: 5' TTTCATGGACCACCAGTGTGC 3'
SOD2	6648	F: 5' GTGGAGAACCCAAAGGGGAGTT 3'<br>R: 5' TTTCATGGACCACCAGTGTGC 3'
GSR	2936	F: 5' TCACGCAGTTACCAAAAGGAAA 3'<br>R: 5' CACACCCAAGTCCCCTGCATAT 3'
CAT	847	F: 5' GTTACTCAGGTGCGGGCATTCTAT 3'<br>R: 5' GAAGTTCTTGACCGCTTTCTTCTG 3'
NFE2L2	4780	F: 5´-CAGCGACGGAAAGAGTATGA-3<br>R: 5´-TGGGCAACCTGGGAGTAG-3´

Abbreviations: F, foward sequence; R, reverse sequence
