# Supplementary material for: Gene expression evaluation of antioxidant enzymes in patients with hepatocellular carcinoma: RT-qPCR and bioinformatic analyses
Source: Genet Mol Biol. 2021 Apr 2;44(2):e20190373. doi: 10.1590/1678-4685-GMB-2019-0373 (PMC8022359; doi:10.1590/1678-4685-GMB-2019-0373)
Supplement: Table S2 - [file 1415-4757-GMB-44-2-e20190373-s2.pdf]

*Genetics and Molecular Biology*
https://doi.org/10.1590/1678-4685-GMB-2019-0373

# Supplementary Material to "Gene expression evaluation of antioxidant enzymes in patients with hepatocellular carcinoma: RT-qPCR and bioinformatic analyses"

**Table S2 -** Detailed results of gene expression analysis from RNAseq data.

| Genes | Log2FC | FC | Wald statistic | BH adjusted p-values |
|---|---|---|---|---|
| **TCGA: tumoral x normal adjacent tissues** | | | | |
| ***GPX1*** | 0.3616 | 1.2849 | 2.3186 | 0.0417 |
| ***GPX4*** | 0.2418 | 1.1825 | 1.6216 | 0.1691 |
| ***SEP15*** | 0.0291 | 1.0203 | 0.3219 | 0.8125 |
| ***SELENOP*** | -0.7322 | 0.6020 | -4.3953 | <0.001 |
| ***SOD1*** | -0.5405 | 0.6875 | -3.4356 | 0.0018 |
| ***SOD2*** | -0.4753 | 0.7193 | -2.6170 | 0.0201 |
| ***GSR*** | 0.1840 | 1.1360 | 1.2959 | 0.2818 |
| ***CAT*** | -1.2491 | 0.4207 | -7.7742 | <0.001 |
| ***NFE2L2*** | -0.6041 | 0.6579 | -5.5939 | <0.001 |
| **TCGA (case) x GTEx (control)** | | | | |
| ***GPX1*** | 0.5181 | 1.432 | 6.3326 | 5.60E-10 |
| ***GPX4*** | 0.6792 | 1.601 | 10.4244 | 8.39E-25 |
| ***SEP15*** | 0.4108 | 1.329 | 8.9075 | 1.77E-18 |
| ***SELENOP*** | 0.2953 | 1.227 | 2.8004 | 0.0074 |
| ***SOD1*** | 0.0079 | 1.005 | 0.0917 | 0.9362 |
| ***SOD2*** | -0.6257 | 0.648 | -5.0087 | 1.06E-06 |
| ***GSR*** | 0.5010 | 1.415 | 6.1414 | 1.85E-09 |
| ***CAT*** | -0.2278 | 0.854 | -2.2139 | 0.0366 |
| ***NFE2L2*** | 0.2046 | 1.152 | 3.5121 | 0.0007 |

Log2FC, log2 Fold-change; FC, fold-change; BH, bonferroni hochberg correction. Significant p-value <0.05
