# Supplementary material for: Gene expression evaluation of antioxidant enzymes in patients with hepatocellular carcinoma: RT-qPCR and bioinformatic analyses
Source: Genet Mol Biol. 2021 Apr 2;44(2):e20190373. doi: 10.1590/1678-4685-GMB-2019-0373 (PMC8022359; doi:10.1590/1678-4685-GMB-2019-0373)
Supplement: Table S3 - [file 1415-4757-GMB-44-2-e20190373-s3.pdf]

Genetics and Molecular Biology
https://doi.org/10.1590/1678-4685-GMB-2019-0373

Supplementary Material to "Gene expression evaluation of antioxidant enzymes in patients with hepatocellular carcinoma: RT-qPCR and bioinformatic analyses"

Table S3 - Final models of Multivariate Cox proportional hazards regression analysis (Backward – stepwise method) for each gene.

	Adjusted HR	95% CI	p
GPX1			
Sex	1.685	0.986-2.881	0.056
Age	1.017	0.999-1.035	0.069
GPX1 expression	2.023	1.222-3.347	0.006
GPX4			
Histologic grade	0.595	0.355-0.997	0.49
Pathologic tumor	1.498	0.936-2.396	0.92
GPX4 expression	1.859	1.147-3.015	0.012
SELENOP			
Histologic grade	0.531	0.312-0.905	0.2
Pathologic tumor	1.539	0.964-2.456	0.071
SELENOP expression	1.968	1.217-3.183	0.006
SOD1			
Sex	1.54	0.913-2.597	0.105
Age	1.015	0.998-1.033	0.089
Histologic grade	0.652	0.387-1.097	0.107
Pathologic tumor	1.691	1.059-2.699	0.028
SOD1 expression	2.447	1.273-4.702	0.007
GSR			
Histologic grade	0.584	0.350-0.975	0.04
Pathologic tumor	1.576	0.989-2.512	0.056
GSR expression	0.354	0.224-0.559	<0.001
CAT			
Histologic grade	0.502	0.295-0.855	0.011
Pathologic tumor	1.516	0.950-2.421	0.081
CAT expression	2.924	1.774-4.820	<0.001
NFE2L2			
Age	1.016	0.998-1.033	0.078
Pathologic tumor	1.649	1.031-2.637	0.037
NFE2L2 expression	2.955	1.779-4.908	<0.001

HR: hazard ratio; CI: confidence interval
