# Supplementary material for: Gene expression evaluation of antioxidant enzymes in patients with hepatocellular carcinoma: RT-qPCR and bioinformatic analyses
Source: Genet Mol Biol. 2021 Apr 2;44(2):e20190373. doi: 10.1590/1678-4685-GMB-2019-0373 (PMC8022359; doi:10.1590/1678-4685-GMB-2019-0373)
Supplement: Figure S1 - [file 1415-4757-GMB-44-2-e20190373-s4.pdf]

Genetics and Molecular Biology
https://doi.org/10.1590/1678-4685-GMB-2019-0373

Supplementary Material to "Gene expression evaluation of antioxidant enzymes in patients with hepatocellular carcinoma: RT-qPCR and bioinformatic analyses"

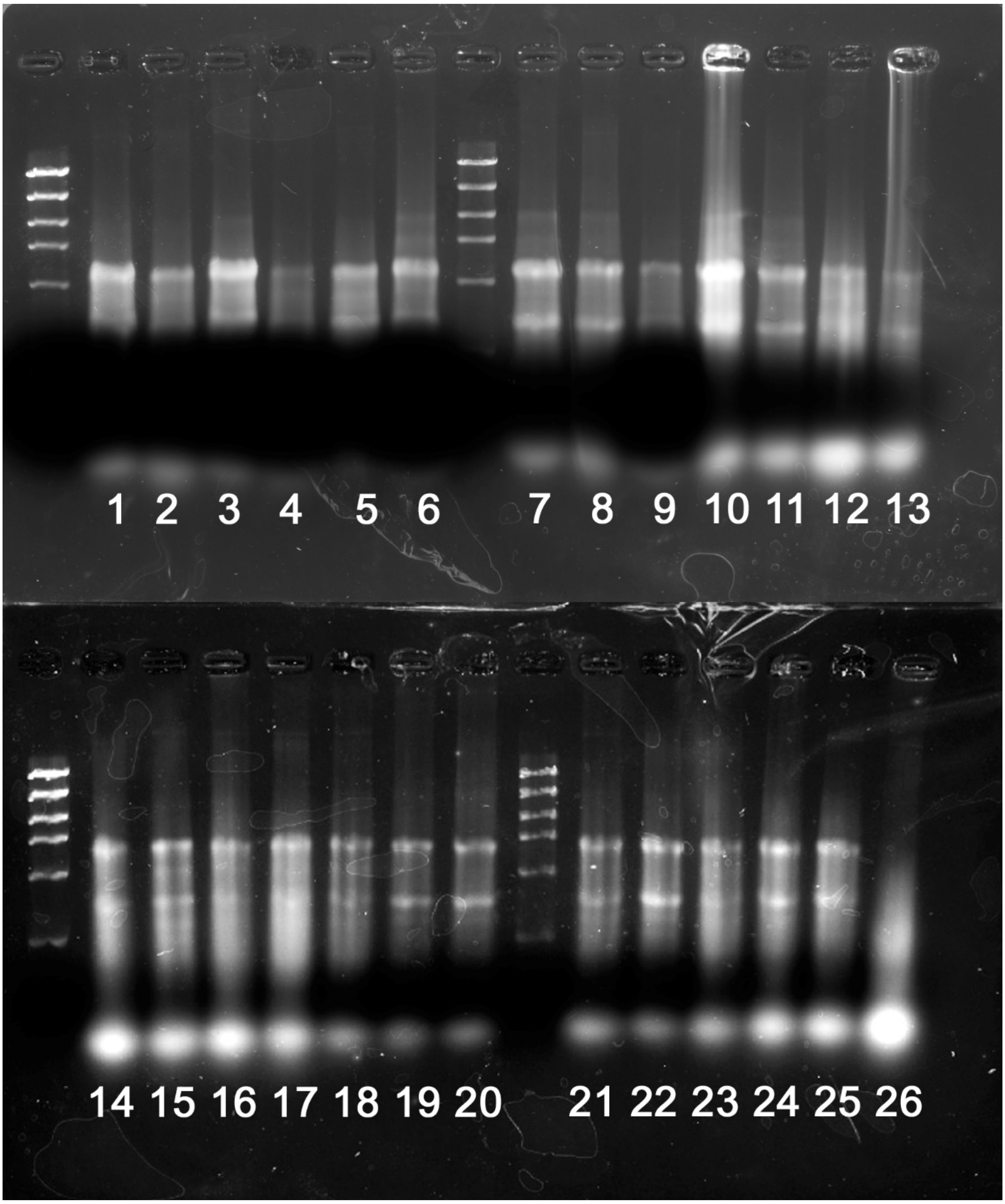

Figure S1 - Sample of agarose gel electrophoresis of total RNA from tumor and peritumoral samples of patients from ISCMPA. Integrity was assessed by the presence of bands corresponding to the 28S and 18S rRNA subunits. Odd numbers represent peritumoral samples and whole numbers represent tumoral samples.
